# Global high-resolution emissions of soil NO_x_, sea salt aerosols, and biogenic volatile organic compounds

**DOI:** 10.1038/s41597-020-0488-5

**Published:** 2020-05-20

**Authors:** Hongjian Weng, Jintai Lin, Randall Martin, Dylan B. Millet, Lyatt Jaeglé, David Ridley, Christoph Keller, Chi Li, Mingxi Du, Jun Meng

**Affiliations:** 1 Laboratory for Climate and Ocean-Atmosphere Studies, Department of Atmospheric and Oceanic Sciences, School of Physics, Peking University, Beijing, 100871 China; 2 Energy, Environmental & Chemical Engineering, Washington University in St. Louis, St. Louis, 63130 MO USA; 3 Department of Soil, Water, and Climate, University of Minnesota Twin Cities, St Paul, MN 55455 USA; 4 Department of Atmospheric Sciences, University of Washington, Seattle, WA 98195 USA; 5 Department of Civil and Environmental, Massachusetts Institute of Technology, Cambridge, MA 02139 USA; 6 Goddard Space Flight Center, NASA Global Modeling and Assimilation Office, Greenbelt, MD 20771 USA; 7 Department of Physics and Atmospheric Science, Dalhousie University, Halifax, NS B3H 4R2 Canada

**Keywords:** Environmental sciences, Atmospheric science

## Abstract

Natural emissions of air pollutants from the surface play major roles in air quality and climate change. In particular, nitrogen oxides (NO_x_) emitted from soils contribute ~15% of global NO_x_ emissions, sea salt aerosols are a major player in the climate and chemistry of the marine atmosphere, and biogenic emissions are the dominant source of non-methane volatile organic compounds at the global scale. These natural emissions are often estimated using nonlinear parameterizations, which are sensitive to the horizontal resolutions of inputted meteorological and ancillary data. Here we use the HEMCO model to compute these emissions worldwide at horizontal resolutions of 0.5° lat. × 0.625° lon. for 1980–2017 and 0.25° lat. × 0.3125° lon. for 2014–2017. We further offer the respective emissions at lower resolutions, which can be used to evaluate the impacts of resolution on estimated global and regional emissions. Our long-term high-resolution emission datasets offer useful information to study natural pollution sources and their impacts on air quality, climate, and the carbon cycle.

## Abstract (summary)

**Table:** 

Measurement(s)	nitrogen oxide • aerosol • isoprene • acetone • acetaldehyde • ethene • ethanol • propene • monoterpene • lumped monoterpenes • limonene • sesquiterpene • Emission • sea salt aerosol
Technology Type(s)	computational modeling technique
Factor Type(s)	spatial resolution • temporal resolution
Sample Characteristic - Environment	saline aerosol environment • soil environment • atmospheric ozone • vegetation layer • atmosphere
Sample Characteristic - Location	Earth (planet)

Machine-accessible metadata file describing the reported data: 10.6084/m9.figshare.12205379

## Background & Summary

Emissions of air pollutants from surface natural processes are an essential component of the Earth system, with strong impacts on air quality, climate and ecosystems. In particular, soil emissions of nitrogen oxides (soil NO_x_) contributed ~50% of global NO_x_ emissions in preindustrial times and currently contribute ~15%, and are a major source of the NO_x_ budget outside of cities^1–3^. Sea salt aerosols (SSAs) are a key player in the climate and chemistry of the marine atmosphere, and dominate the top-of-atmosphere clear sky radiative effect over the oceans^4–6^. They are also an important source of halogens, provide large surface area for heterogeneous reactions, and affect ozone, nitrogen, bromine chemistry and many other pollutants^7–12^. Biogenic non-methane volatile organic compounds (BVOCs), among which the most abundant species are isoprene and monoterpenes, are the dominant contributor to the global VOCs flux into the atmosphere^13–15^. BVOCs affect the production of near-surface ozone in urban and surrounding areas^16,17^, alter the atmospheric oxidative capability and methane lifetime on regional and global scales^18,19^, and are important precursors of carbon dioxide^14,20^.

Natural emissions are nonlinearly dependent on meteorological factors such as temperature, radiation, humidity, and winds^2,6,14,21^. For soil NO_x_ and BVOCs, the properties of soils (e.g., water content, organics contents, microbes, and the amount of fertilizer applied) and vegetation (e.g., type, density and physiology) are also critical^2,14,15,22^. Especially for the global domain, these natural emissions are typically estimated through parameterization, due to inadequate mechanistic knowledge about emission processes as well as concerns about the computational costs needed to fully resolve such processes. Parameterizations are typically nonlinear – meaning that the horizontal resolution of inputted meteorological and other variables has an important influence on the calculated emission totals and spatial distributions. Parameterizations are typically embedded in three-dimensional (3-D) chemical transport models (CTMs), climate-chemistry models and earth system models to calculate natural emissions online, and are thus sensitive to model resolution. Resolution-dependent emissions are a major factor affecting the accuracy of 3-D models^23–27^.

Moreover, historical records of global high-resolution (≤50 km) natural emissions, shown in Tables 1–3, are relatively small, hindering the understanding of variations in global emission totals, spatial distributions and their air quality and climate impacts.

**Table 1:** Comparison with previous studies for soil NO_x_ emissions.

Reference	Time	Method	Resolution	Emission (Tg N/yr)
**Top-down**				
Miyazaki, *et al*.^47^	2005–2014	Multi-constituent satellite data assimilation	2.5° lat. × 2.5° lon.	7.9
Vinken, *et al*.^3^	2005	OMI, GEOS-Chem	2° lat. × 2.5° lon.	9–16.8
Stavrakou, *et al*.^48^	2007	DOMINO, IMAGES CTM, MINLOSS setting	2° lat. × 2.5° lon.	9
Stavrakou, *et al*.^48^	2007	DOMINO, IMAGES CTM, MAXLOSS setting	2° lat. × 2.5° lon.	20.4
Stavrakou, *et al*.^49^	1997–2006	GOME, SCIAMACHY, IMAGES CTM	2° lat. × 2.5° lon.	10–12
Jaeglé, *et al*.^50^	2000	GOME, GEOS-Chem	2° lat. × 2.5° lon.	7–10.8
Müller and Stavrakou^51^	1997	GOME, IMAGES	5° lat. × 5° lon.	10.9–12.1
**Bottom-up**				
Heald, *et al*.^52^	2000	BNSNP	0.5° lat. × 0.667° lon.	10
Hudman, *et al*.^2^	2006	YL95	2° lat. × 2.5° lon.	6.2
Hudman, *et al*.^2^	2006	BNSNP	2° lat. × 2.5° lon.	9
Steinkamp and Lawrence^30^	1990–2000	YL95EMAC	~1.1° lat. × 1.1° lon.	8.6
Yan, *et al*.^34^	2001	Statistical model	0.5° lat. × 0.5° lon.	5
Ganzeveld, *et al*.^53^	2000	YL95	~3.75° lat. × 3.75° lon.	8
Yienger and Levy II^43^	1990	YL95	2° lat. × 2.5° lon.	3.3–7.7
Müller^54^	1980	Chemical transport model	5° lat. × 5° lon.	4.1
This Study	1980–2017	Updated BNSNP, MERRA-2	0.5° lat. × 0.625° lon.	9.5
This Study	2014–2017	Updated BNSNP, GEOS-FP	4° lat. × 5° lon.	7.1
This Study	2014–2017	Updated BNSNP, GEOS-FP	2° lat. × 2.5° lon.	7.5
This Study	2014–2017	Updated BNSNP, GEOS-FP	0.25° lat. × 0.3125° lon.	8.8

**Table 2:** Comparison with previous studies for sea salt emissions.

Reference	Year	Method	Resolution	Emission (Tg/yr)
Zhu, *et al*.^12^	2011–2012	Statistical model (Jaeglé, *et al*.^6^) in GEOS-Chem 12.3.0	4° lat. × 5° lon.	3,140
Grythe, *et al*.^55^	1980–2005	FLEXPART	1° lat. × 1° lon.	8,910
Grythe, *et al*.^55^	1980–2005	FLEXPART fit to the observed	1° lat. × 1° lon.	9,000–10,800
Sofiev, *et al*.^56^	2001; 2008	SILAM	1° lat. × 1° lon.	7,050
Jaeglé, *et al*.^6^ MODEL-STD	2008	Statistical model (Gong^39^)	2° lat. × 2.5° lon.	5,200
Jaeglé, *et al*.^6^ MODEL-SST	2008	Statistical model (Jaeglé, *et al*.^6^)	2° lat. × 2.5° lon.	4,600
This Study	1980–2017	Statistical model (Jaeglé, *et al*.^6^) in HEMCO, MERRA-2	0.5° lat. × 0.625° lon.	3,560
This Study	2014–2017	Statistical model (Jaeglé, *et al*.^6^) in HEMCO, GEOS-FP	4° lat. × 5° lon.	3,156
This Study	2014–2017	Statistical model (Jaeglé, *et al*.^6^) in HEMCO, GEOS-FP	2° lat. × 2.5° lon.	3,239
This Study	2014–2017	Statistical model (Jaeglé, *et al*.^6^) in HEMCO, GEOS-FP	0.25° lat. × 0.3125° lon.	3,860

**Table 3:** Comparison with previous studies for biogenic isoprene emissions.

Reference	Year	Method	Resolution	Emission (Tg C/yr)
**Top-down**				
Bauwens, *et al*.^17^	2005–2013	OMI-based	0.5° lat. × 0.5° lon.	240
Shim, *et al*.^46^	1996–1997	GOME, GEOS-Chem	4° lat. × 5° lon.	566
**Bottom-up**				
Henrot, *et al*.^57^	2000–2012	MEGAN v2.1 in ECHAM6-HAMMOZ	1.875° lat. × 1.875° lon.	417
Bauwens, *et al*.^17^	2005–2013	MEGAN-MOHYCAN	0.5° lat. × 0.5° lon.	303
Bauwens, *et al*.^17^	2005–2009	GUESS-ES	1° lat. × 1° lon.	399
Messina, *et al*.^58^	2000–2009	MEGAN v2.1	0.5° lat. × 0.5° lon.	428
Messina, *et al*.^58^	2000–2009	MEGAN v2.1 in ORCHIDEE	0.5° lat. × 0.5° lon.	465
Sindelarova, *et al*.^15^	1980–2010	MEGAN-MACC	0.5° lat. × 0.5° lon.	524
Guenther, *et al*.^14^	2000	MEGAN v2.1 in CLM	0.5° lat. × 0.5° lon.	472
Arneth, *et al*.^45^	1981–2002	LPJ-GUESS	0.5° lat. × 0.5° lon.	463
Arneth, *et al*.^45^	1981–2002	MEGAN v2	0.5° lat. × 0.5° lon.	378
Arneth, *et al*.^45^	1981–2002	BVOCEM	0.5° lat. × 0.5° lon.	471
Emmons, *et al*.^59^	2000–2007	MEGAN v2 in MOZART4	2.8° lat. × 2.8° lon.	414
Young, *et al*.^60^	2008	LPJ-GUESS	2.5° lat. × 3.75° lon.	401
Pfister, *et al*.^16^	2005	MEGAN v2 in MOZART4	4° lat. × 5° lon.	470
Müller, *et al*.^61^	1995–2006	MEGAN-MOHYCAN	0.5° lat. × 0.5° lon.	361
Wiedinmyer, *et al*.^62^	1990–2000	MEGAN	0.5° lat. × 0.5° lon.	461
Guenther, *et al*.^63^	2003	MEGAN v2	0.5° lat. × 0.5° lon.	529
Naik, *et al*.^64^	1961–1990	IBIS v2.5	2° lat. × 2° lon.	454
Levis, *et al*.^65^	1990	MEGAN in CLM	1° lat. × 1° lon.	507
Guenther, *et al*.^66^	1990	MEGAN	0.5° lat. × 0.5° lon.	503
This Study	1980–2017	MEGAN v2.1 in HEMCO, MERRA-2	0.5° lat. × 0.625° lon.	345
This Study	2014–2017	MEGAN v2.1 in HEMCO, GEOS-FP	4° lat. × 5° lon.	330
This Study	2014–2017	MEGAN v2.1 in HEMCO, GEOS-FP	2° lat. × 2.5° lon.	333
This Study	2014–2017	MEGAN v2.1 in HEMCO, GEOS-FP	0.25° lat. × 0.3125° lon.	341

Here we use the Harvard-NASA Emissions Component^28^ (HEMCO) to produce monthly global emissions of soil NO_x_, SSAs, and BVOCs at different resolutions. These emissions are calculated at 0.5° lat. × 0.625° lon. for 1980–2017 using the MERRA-2 assimilated meteorology and at three resolutions (0.25° lat. × 0.3125° lon., 2° lat. × 2.5° lon., and 4° lat. × 5° lon.) for 2014–2017 using GEOS-FP. The datasets will be continuously updated and published. The datasets can be used to study the effects of these natural emissions on air quality, climate, and the carbon cycle, as well as the effects of horizontal resolution on emissions estimates. The datasets can be downloaded freely through Peking University Atmospheric Chemistry & Modeling Group (http://www.phy.pku.edu.cn/~acm/acmProduct.php#NATURAL-EMISSION) and Figshare^29^.

## Methods

### HEMCO

The HEMCO^28^ is a software package to compute pollutant emissions at user-defined resolutions. HEMCO can be run in a standalone mode or coupled to a 3-D model like GEOS-Chem. Here we use HEMCO version 2.1 at the standalone mode to calculate natural emissions based on different meteorological, ancillary variables, and nonlinear parameterizations.

### Soil NO_x_ emissions

Inside HEMCO, the algorithm for above-canopy soil NO_x_ emissions (soil NO_x_) follows Hudman, *et al*.^2^, with the efficiency of loss to canopy depending on vegetation type and density. Based on soil chamber and field measurements, soil NO_x_ varies greatly with climate and edaphic conditions, and are most strongly correlated with N-availability, temperature, precipitation patterns, and fertilizer management practices^21,22^. In the Hudman, *et al*.^2^ algorithm, soil NO_x_ emissions flux is a complex function of biological and meteorological drivers:

1 $${S}_{N{O}_{x}}={A{\prime} }_{biome}({N}_{avail})\times f\left(T\right)\times g\left(\theta \right)\times P({l}_{dry},t)$$

2 $${A{\prime} }_{biome}={A}_{w,biome}+{N}_{avail}\times \bar{\mathrm{E}}$$

3 $${N}_{avail}\left(t\right)={N}_{avail}\left(0\right){e}^{-\frac{t}{\tau }}+F\times \tau \times \left(1-{e}^{-\frac{t}{\tau }}\right)$$

4 $$f\left(T\right)\times g\left(\theta \right)={e}^{0.103T}\times a\theta {e}^{-b{\theta }^{2}}$$

5 $$P\left({l}_{dry},t\right)=\left[13.01\,{\rm{ln}}({l}_{dry})-53.6\right]\times {e}^{-ct}$$

${A{\prime} }_{biome}$, representing the biome-dependent emission factors of N in the soil, is a function of ${N}_{avail}$ and the ${A}_{w,biome}$ coefficients. ${A}_{w,biome}$ is the wet biome-dependent emission factors updated based on estimates from Steinkamp and Lawrence^30^. $\bar{\mathrm{E}}$ is the mean emission rate of fertilizer, and is treated identically to the natural pool of N.

${N}_{avail}$, representing the sum of fertilizer N and deposited N, is the mass of available nitrogen in the soil. *F* is the fertilizer application rate and *τ* is a decay lifetime, which is chosen as 4 months based on measurements within the top 10 cm of soil^31,32^. Although atmospheric deposition also contributes to the available nitrogen in soils (about ~5% globally, based on Hudman, *et al*.^2^), this amount can only be calculated through 3-D model simulations and is thus not accounted for here.

$f(T)\times g(\theta )$ represents the combination of the soil temperature (*T*) and soil moisture dependence of soil NO_x_. The temperature dependence of soil NO_x_ is an exponential dependence on temperature between 0 °C and 30 °C (constant at *T* > 30 °C), where 0.103 is the weighted average of temperature dependencies for several biomes. The parameterization for soil moisture is a Poisson function scaling, where *θ* (water-filled pore space) is defined as the ratio of the volumetric soil moisture content to the porosity^33^.

$P({l}_{dry},t)$ represents the pulsed soil NO_x_, which occur when very dry soil is wetted resulting in a reactivation of water-stressed bacteria. The parameterization, following Yan, *et al*.^34^ is derived from four field studies relating pulsed emissions to the length of the antecedent dry period^35–38^. The rate constant *c* reflects the rise/fall time of the pulse (*c* = 0.068 h^−1^). The value of *l*_*dry*_ is the antecedent dry period in hours.

### Emissions of sea salt aerosols

Parametrization of sea salt emissions in HEMCO is modified from Jaeglé, *et al*.^6^. It considers two categories of SSAs based on their radii. The radius of accumulation mode sea salt aerosol (SALA) ranges from 0.01 to 0.5 μm, while that for coarse mode sea salt aerosol (SALC) ranges from 0.5 to 8 μm.

Parametrization of sea salt aerosols emissions includes both a wind speed and a sea surface temperature (SST) dependence. The SSAs emission flux density function dE/dr_80_ is formulated as follows:

6 $$\frac{{\rm{dE}}}{{\rm{d}}{r}_{80}}=\left(0.3+0.1T-0.0076{T}^{2}+0.00021{T}^{3}\right)\times 1.373{u}_{10m}^{3.41}{r}_{80}^{-A}\times \left(1+0.057{r}_{80}^{3.45}\right)\times 1{0}^{1.607{e}^{-{B}^{2}}}$$

The SST dependence $\left(0.3+0.1T-0.0076{T}^{2}+0.00021{T}^{3}\right)$ was derived based on a comparison of the GEOS-Chem sea salt simulation with SALC mass concentration observations obtained from six cruises conducted by the National Oceanic and Atmospheric Administration Pacific Marine Environmental Laboratory^6^.

The sea salt source function $\left(1.373{u}_{10m}^{3.41}{r}_{80}^{-A}\times \left(1+0.057{r}_{80}^{3.45}\right)\times 1{0}^{1.607{e}^{-{B}^{2}}}\right)$ is based on Gong^39^. *r*_80_ is the particle radius at RH = 80% (with r_80_ ~ 2r_dry_), and *u*_10m_ is the 10-meter wind speed. ${\rm{A}}=4.7{(1+\Theta {r}_{80})}^{-0.017{r}_{80}^{-1.44}}$, and ${\rm{B}}=\left[0.433-{{\rm{\log }}}_{10}\left({r}_{80}\right)\right]/0.433$. The adjustable parameter Θ controls the shape of the size distribution for submicron aerosols, and the value we use is 30 according to Gong^39^.

### Biogenic VOC emissions

Inside HEMCO, BVOCs emissions are computed by the Model of Emissions of Gases and Aerosols from Nature version 2.1 (MEGAN2.1)^14^. MEGAN2.1 includes two major components: calculation of landscape average emission factors, and algorithms describing emission responses to variations in environmental conditions. The emissions (*F*_*i*_) of species *i* is the product of the two components summed over all vegetation types:

7 $${F}_{i}={\gamma }_{i}\sum {\varepsilon }_{i,j}{\chi }_{j}$$

8 $${\gamma }_{i}=CL{\gamma }_{p,i}{\gamma }_{T,i}{\gamma }_{A,i}{\gamma }_{S,i}{\gamma }_{C,i}$$

${\varepsilon }_{i,j}$ is the average emission factor of species *i* for vegetation type *j* at standard conditions (leaf temperature = 297 K; air temperature = 303 K; the photosynthetic photon flux density averaged over the past 24 h is equal to 200 μmol m^−2^s^−1^ for sun leaves and 50 μmol m^−2^s^−1^ for shade leaves), and *χ*_*j*_ is the fractional grid box areal coverage for the same vegetation type. The emission factor accounts for the estimated in-canopy deposition flux so that *F*_*i*_ represents the net above-canopy flux.

The emission activity factor (*γ*_*i*_) reflects the emission response to environmental drivers. The canopy environment coefficient (*C*) is assigned a value that results in γ = 1 for the standard conditions and is dependent on the canopy environment model being used. A detailed description of the model parameterizations for light (*γ*_*p*_), temperature (*γ*_*T*_), leaf age (*γ*_*A*_), soil moisture (*γ*_*S*_), leaf area index (*L*) and CO_2_ inhibition (*γ*_*C*_) can be obtained from Guenther^14^.

Our BVOCs emission dataset includes isoprene (ISOP, the most abundant species), acetone (ACET), acetaldehyde (ALD_2_), ethene (C_2_H_4_), ethanol (EOH), propene (PRPE), lumped monoterpenes (MTPA, sum of α pinene, β pinene, sabinene and carene), other monoterpenes (MTPO, sum of myrcene, ocimene and other monoterpenes), limonene (LIMO), and sesquiterpenes (SESQ, sum of farnesene, β caryoph and other sesquiterpenes).

### LAI data for calculation of soil NO_x_ and BVOCs emissions

Vegetation composition is principal information needed to estimate BVOCs and soil NO_x_ emissions^14,15^. The density of vegetation is represented in the parametrizations by leaf area index (LAI), which is defined as the amount of leaf area per unit surface of the ground (*m*^2^*m*^−2^). We use monthly MODIS-derived LAI with gap filling and smoothing described by Yuan, *et al*.^40^ (here after referred to as Yuan LAI). For 2005–2017, we use year-specific Yuan LAI data. For years prior to 2005, we use the LAI values in 2005 due to lack of year-specific data. This would introduce certain uncertainty for these earlier years. We did a test to fix LAI to a certain year, and the effect on global emissions is relatively small (within 5%). Therefore, our extrapolation of LAI data before 2005 does not significantly affect the emissions time series.

## Data Records

Our datasets contain 4 data records for monthly global gridded emissions. Each record contains monthly emission data for soil NO_x_, 2 SSAs species (SALA and SALC), and 10 BVOCs species (ISOP, ACET, ALD_2_, C_2_H_4_, EOH, PRPE, MTPA, MTPO, LIMO, and SESQ). Our data were constructed in nc file format which can be read by many tools like IDL, MatLab, and so on. Of these,

- One is from 1980 to 2017 based on MERRA-2 at 0.5° lat. × 0.625° lon. [Available in Hongjian and Jintai^29^, File ‘MERRA-2_05 × 0625_monthly_1980–2017.nc’];
- One is from 2014 to 2017 based on GEOS-FP at 0.25° lat. × 0.3125° lon. [Available in Hongjian and Jintai^29^, File ‘GEOS-FP_025 × 03125_monthly_2014–2017.nc’];
- One is from 2014 to 2017 based on GEOS-FP at 2° lat. × 2.5° lon. [Available in Hongjian and Jintai^29^, File GEOS-FP_2 × 25_monthly_2014–2017.nc’];
- One is from 2014 to 2017 based on GEOS-FP at 4° lat. × 5° lon. [Available in Hongjian and Jintai^29^, File ‘GEOS-FP_4 × 5_monthly_2014–2017.nc’];

Table 4 presents the global annual total emissions of soil NO_x_, SSAs, and BVOCs over 1980–2017 derived from MERRA-2 at 0.5° lat. × 0.625° lon. Averaged over all years, global total soil NO_x_ emissions amounts to 9.5 TgN/yr. Global total SSAs reaches 3,560 Tg/yr, as contributed by SALC (98.4%) and SALA (1.6%). Global total BVOCs emission reaches 563 TgC/yr, as contributed by emissions of ISOP (61.2%), ACET (4.4%), ALD_2_ (1.6%), C_2_H_4_ (3.4%), EOH (1.6%), PRPE (3.0%), MTPA (13.5%), MTPO (6.4%), LIMO (1.5%), and SESQ (3.4%).

**Table 4:** Global annual total emissions of soil NO_x_, SSAs, and BVOCs (with standard deviation) over 1980–2017 derived based on MERRA-2 at 0.5° lat. × 0.625° lon.

Species		1980–2017 mean	Rel. contribution	Maximum	Minimum
Soil NO_x_ (TgN/year)		9.5 ± 0.4	100%	10.5	8.5
Sea salt aerosols (Tg/year)	Accumulation mode	57.9 ± 2.6	1.6%	62.3	52.0
	Coarse mode	3,502 ± 157	98.4%	3,771	3,150
Biogenic VOCs (TgC/year)	Isoprene	345 ± 17	61.2%	381	315
	Acetone	24.7 ± 0.9	4.4%	26.6	23.1
	Acetaldehyde	9.2 ± 0.4	1.6%	9.9	8.7
	Ethene	19.1 ± 0.8	3.4%	20.7	17.7
	Ethanol	8.8 ± 0.3	1.6%	9.5	8.3
	Propene	16.9 ± 0.6	3.0%	18.1	15.9
	Lumped monoterpenes	75.7 ± 3.1	13.5%	82.0	70.4
	Other monoterpenes	36.0 ± 1.5	6.4%	39.3	33.3
	Limonene	8.2 ± 0.3	1.5%	8.8	7.7
	Sesquiterpenes	19.1 ± 1.2	3.4%	21.6	16.9

**Fig. 1:**
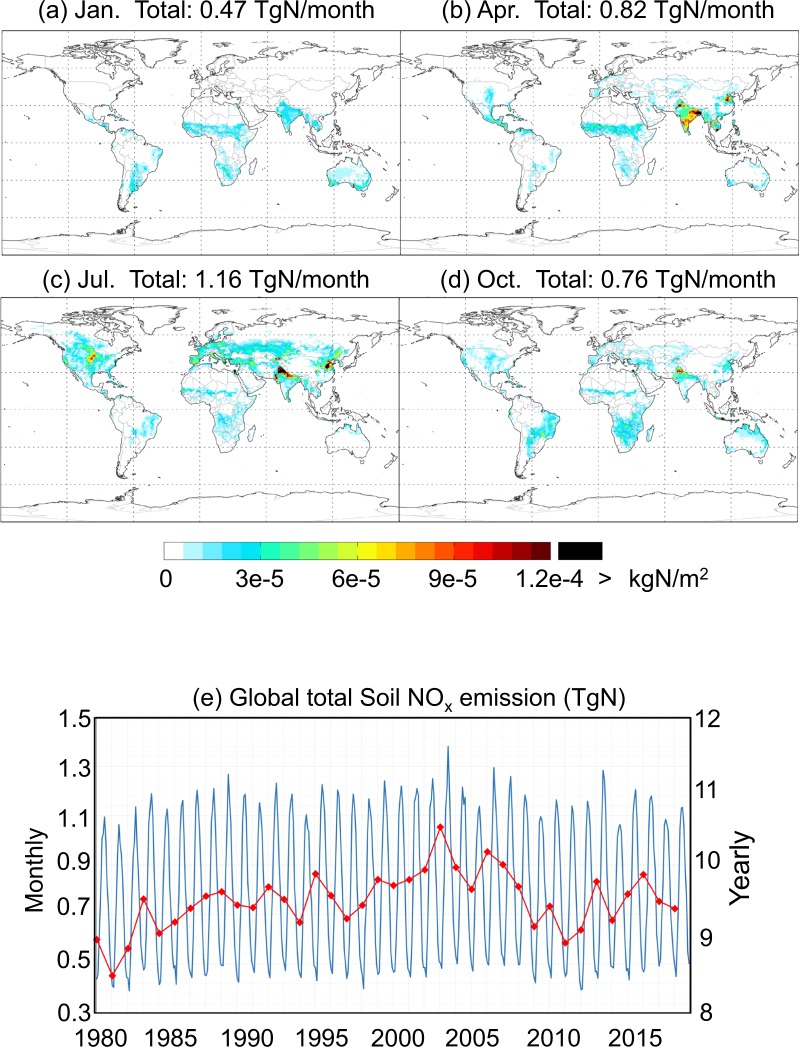
Spatial distribution of soil NO_x_ emissions in January (**a**), April (**b**), July (**c)**, and October (**d**), temporal profile of global monthly total emissions (blue line in e), and temporal profile of global annual total emissions (red line in **e**) over 1980–2017 derived based on MERRA-2 at 0.5° lat. × 0.625° lon.

Figure 1a–d shows the MERRA-2 based spatial distribution of soil NO_x_ emissions at 0.5° lat. × 0.625° lon. in January, April, July, and October averaged over 1980–2017. High values of soil NO_x_ emissions move between the two hemispheres as a result of the seasonal variation of temperature. Spatially, the highest emissions occur over regions with intensive agricultural activities, e.g., the Ganges River Basin of India and the North China Plain. Figure 1e further shows the temporal profile of global monthly total emissions (blue line) and annual total emissions (red line), which indicates the strong seasonality (with a July to January ratio of 2.5) and interannual variability (with maximum values in the early 2000s).

**Fig. 2:**
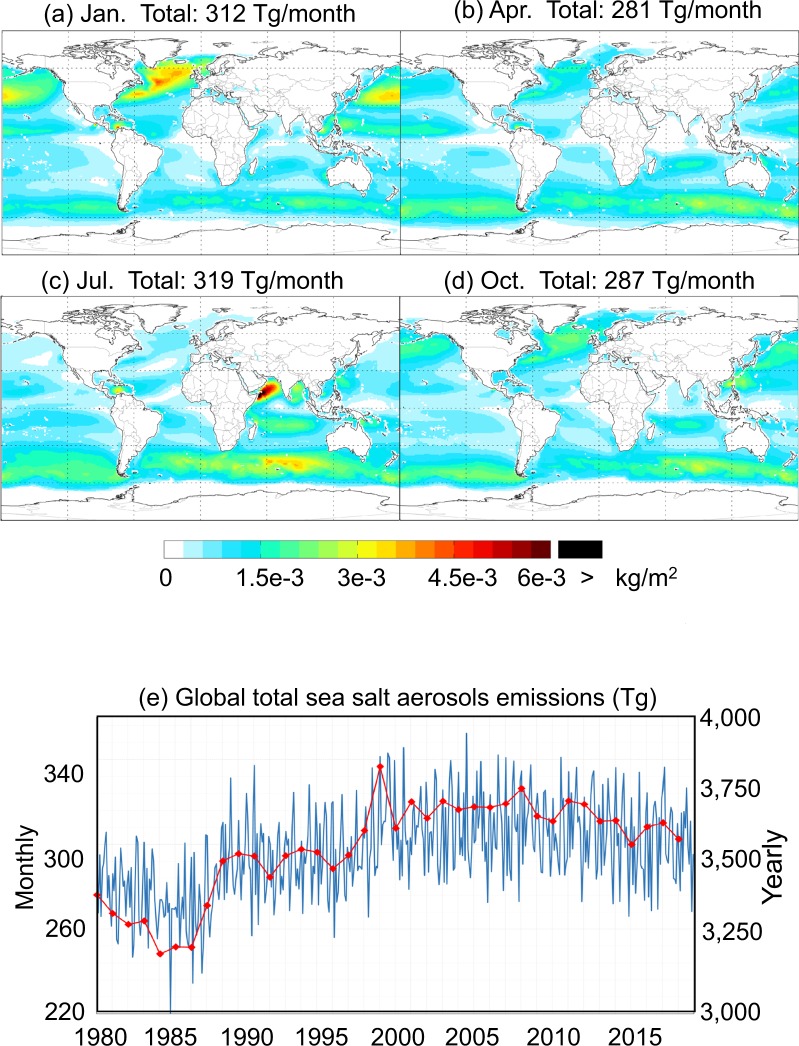
Spatial distribution of SSAs (sum of SALA and SALC) emissions in January (**a**), April (**b**), July (**c**), and October (**d**), temporal profile of global monthly total emissions (blue line in **e**), and temporal profile of global annual total emissions (red line in **e**) over 1980–2017 derived based on MERRA-2 at 0.5° lat. × 0.625° lon.

Figure 2a–d represents the spatial distribution of total SSAs (sum of SALA and SALC) emissions in different seasons, and Fig. 2e shows the temporal profile of the respective global total emissions. SSAs are the largest over the North Atlantic in January and over the Indian Ocean in July. Emissions are also strong over the Southern Ocean. The temporal variation of global total SSAs emissions is characterized by lower values in the 1980s and 1990s than in later years (with a difference by about 10%), and by a modest seasonality (within 15%).

**Fig. 3:**
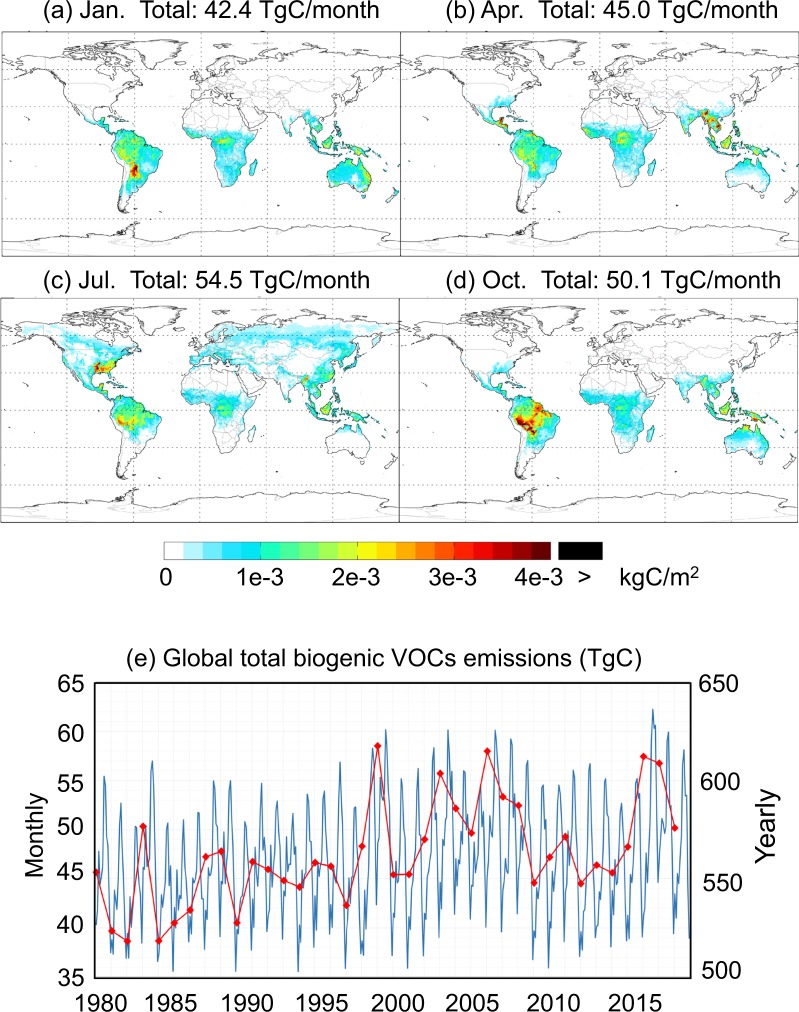
Spatial distribution of total BVOCs (sum of ISOP, ACET, ALD_2_, C_2_H_4_, EOH, PRPE, MTPA, MTPO, LIMO, and SESQ) emissions in January (**a**), April (**b**), July (**c**), and October (**d**), temporal profile of global monthly total emissions (blue line in **e**), and temporal profile of global annual total emissions (red line in **e**) over 1980–2017 derived based on MERRA-2 at 0.5° lat. × 0.625° lon.

Figure 3 shows the spatial and temporal distributions of total BVOCs emissions (sum of ISOP, ACET, ALD_2_, C_2_H_4_, EOH, PRPE, MTPA, MTPO, LIMO, and SESQ). The total BVOCs exhibits strong seasonality and cross-hemispheric seasonal migration (Fig. 3a–d) because of changes in radiation and temperature. The highest emissions occur over the Amazon, Southeast Asia, Southeast United States, and Central Africa. The global total emission also exhibits a large seasonality, with a July to January ratio of 1.3, due to variation of LAI, especially in the Northern Hemisphere. The interannual variation is modest (within 20%) (Fig. 3e).

The parameterized nonlinear relationships between emissions and controlling factors means that the horizontal resolution of inputted meteorological and other variables has important influences on the calculated emission magnitudes and spatial distributions.

Table 5 presents the global annual total emissions of soil NO_x_, SSAs and BVOCs derived based on GEOS-FP at different resolutions (4° lat. × 5° lon., 2° lat. × 2.5° lon., and 0.25° lat. × 0.3125° lon.) over 2014–2017. The resolution dependence of emission magnitude is evident especially for soil NO_x_ and SSAs, that is, a higher resolution results in greater global emission totals. The global total SSAs emission increases from 3,157 Tg/yr to 3,239 Tg/yr (by 2.6%) and to 3,860 Tg/yr (by 22.3%) as the resolution changes from the coarsest to the finest. This increase is primarily because emissions are parameterized as a function of wind speed to the 3.41-th power. For soil NO_x_, the global total increases from 7.1 TgN/yr to 7.5 TgN/yr (by 5.6%) and to 8.8 TgN/yr (by 23.9%) as the resolution increases. This is mainly because the parameterized NO_x_ emission is convex functions of temperature and soil moisture. For BVOCs, the resolution dependence of the global total emission is weaker, i.e., within 5% for ISOP and within 10% for other species. The magnitude of horizontal resolution dependence for BVOCs here is similar to that of temporal resolution dependence shown by Ashworth, *et al*.^41^ who showed that using monthly mean inputted data instead of hourly data would reduce the global ISOP emission total by 7%.

**Table 5:** Global annual total emissions of soil NO_x_, SSAs, and BVOCs over 2014–2017 derived based on GEOS-FP at three resolutions. The percentage values represent the relative changes from emissions at 4° lat. × 5° lon.

Species		4° lat. × 5° lon.	2° lat. × 2.5° lon.	0.25° lat. × 0.3125° lon.
Soil NO_x_ (TgN/year)		7.1	7.5 (+5.6%)	8.8 (+23.9%)
Sea salt aerosols (Tg/year)	Accumulation mode	51.3	52.6 (+2.5%)	62.7 (+22.2%)
	Coarse mode	3,105	3,186 (+2.6%)	3,797 (+22.3%)
Biogenic VOCs (TgC/year)	Isoprene	330	333 (+0.9%)	341 (+3.3%)
	Acetone	27.0	26.2 (−3.0%)	24.8 (−8.1%)
	Acetaldehyde	9.8	9.5 (−3.1%)	9.3 (−5.1%)
	Ethene	20.4	19.9 (−2.5%)	18.9 (−7.4%)
	Ethanol	9.4	9.1 (−3.2%)	8.9 (−5.3%)
	Propene	18.5	18.0 (−2.7%)	17.0 (−8.1%)
	Lumped monoterpenes	80.1	78.6 (−1.9%)	75.7 (−5.5%)
	Other monoterpenes	37.8	37.1 (−1.9%)	35.8 (−5.3%)
	Limonene	8.1	8.2 (+1.2%)	8.3 (+2.5%)
	Sesquiterpenes	20.6	20.0 (−2.9%)	19.2 (−6.8%)

**Fig. 4:**
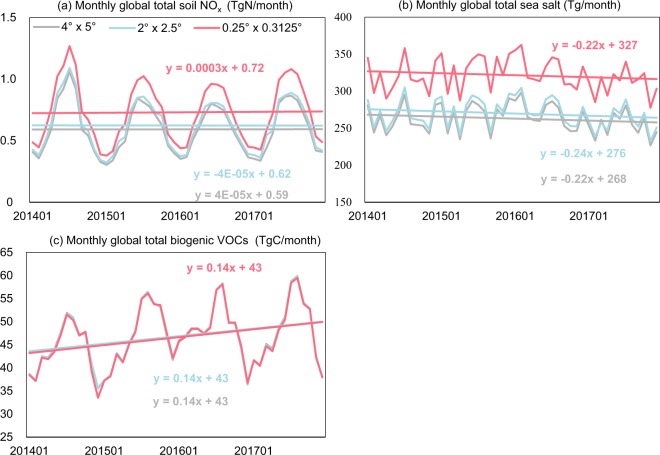
Temporal profiles of monthly global total emissions of soil NO_x_ (**a**), sea salt (**b**) and BVOCs (**c**) over 2014–2017 derived based on GEOS-FP at different resolutions.

Figure 4 shows the temporal profile of monthly global total emissions of soil NO_x_ (a), sea salt (b), and biogenic VOCs (c) over 2014–2017 derived based on GEOS-FP at 4°lat. × 5° lon. (gray line), 2° lat. × 2.5° lon. (blue line), and 0.25° lat. × 0.3125° lon. (red line). Although the total emissions of soil NO_x_ and sea salt increase with the horizontal resolution, the interannual and seasonal variability and trends are similar at different horizontal resolutions.

**Fig. 5:**
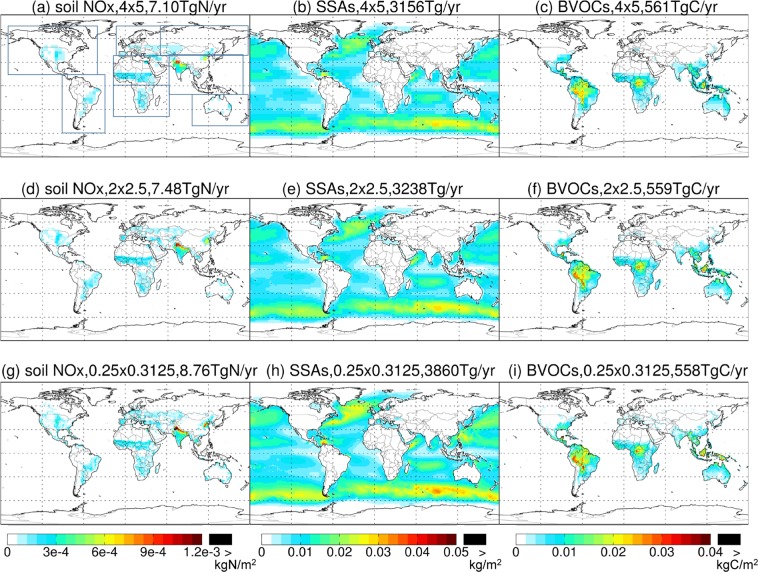
Spatial distributions of annual emissions of soil NO_x_ (first column, kgN/m^2^), SSAs (SALA + SALC, second column, kg/m^2^), and total BVOCs (summed over all species, last column, kgC/m^2^) over 2014–2017 derived based on GEOS-FP at different resolutions. The rectangles in (**a**) show the regions whose regional emission totals are shown in Fig. 9.

**Fig. 6:**
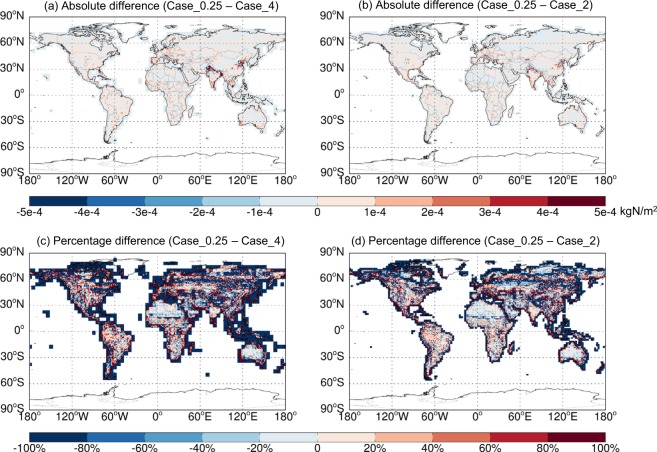
Spatial distributions of annual soil NO_x_ emissions differences between 0.25° lat. × 0.3125° lon. and coarser resolutions over 2014–2017 based on GEOS-FP. Case_0.25 represents emissions at 0.25° lat. × 0.3125° lon. Case_4 represents emissions re-gridded from 4° lat. × 5° lon. to 0.25° lat. × 0.3125° lon. Case_2 represents emissions re-gridded from 2° lat. × 2.5° lon. to 0.25° lat. × 0.3125° lon. Percentage differences are calculated as 2 * (Case_0.25-Case_4)/(Case_0.25 + Case_4)*100% and 2*(Case_0.25-Case_2)/(Case_0.25 + Case_2)*100%.

**Fig. 7:**
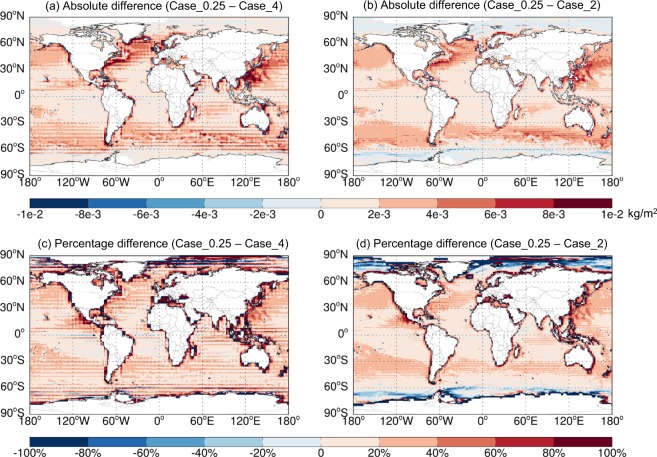
Similar to Fig. 6 but for total sea salt emissions.

**Fig. 8:**
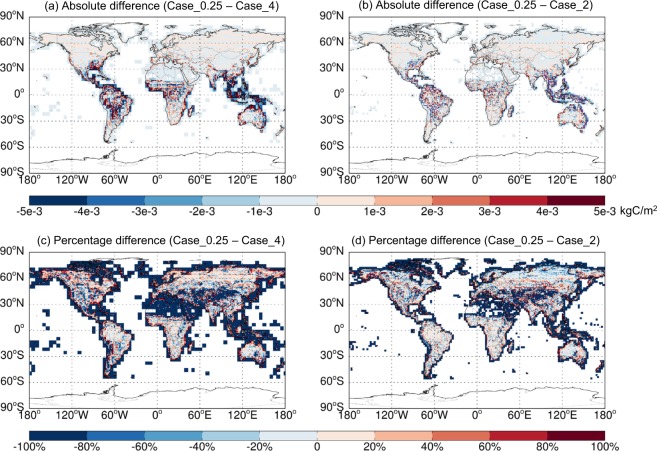
Similar to Fig. 6 but for total BVOCs emissions.

Figure 5 shows the 2014–2017 average spatial distributions of annual emissions of soil NO_x_, SSAs (SALA + SALC), and total BVOCs (summed over all species) estimated at different resolutions based on GEOS-FP. Figures 6–8 further show the respective spatial distributions of emission differences and percentage differences from 0.25° lat. × 0.3125° lon. to coarser resolutions. As the resolution increases, fine scale patterns of emissions become much more evident, which has important implications for air quality simulations. For soil NO_x_, northern India and North China, which are major source regions, exhibit the largest resolution dependence for absolute emission differences (Fig. 6a,b). The percentage difference is most evident along the coasts where a fine resolution (0.25° lat. × 0.3125° lon.) resolves the land-ocean contrast much better than coarser resolutions do (Fig. 6c,d). For sea salt emissions, the major source regions at high latitudes of both hemispheres exhibit a large resolution dependence (Fig. 7). For BVOCs, tropical regions have the largest resolution dependence in terms of absolute difference (Fig. 8a,b), while the coastal and low-emission regions exhibit the largest resolution dependence in terms of percentage difference (Fig. 8c,d).

**Fig. 9:**
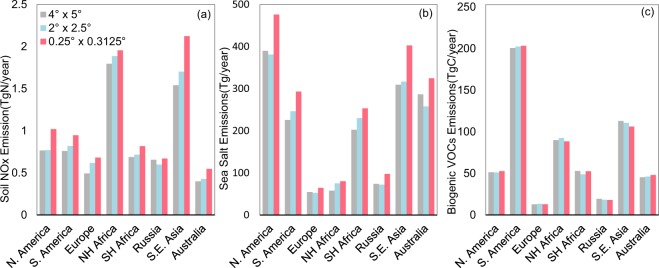
2014–2017 average annual total emissions of soil NO_x_ (**a**), SSAs (SALA + SALC, **b**), and BVOCs (summed over all species, **c**) in eight regions: North America, South America, Europe, Northern Hemisphere Africa, Southern Hemisphere Africa, Russia, Southeast Asia and Australia. See Fig. 5 for regional definitions. Data are derived based on GEOS-FP at different resolutions.

Figure 9 further shows the resolution dependence of calculated regional annual emission totals over eight major regions. Compared to results for global total emissions in Table 5, the resolution dependence of emission magnitude in some regions is more evident. For Southeast Asia, soil NO_x_ emissions total at 0.25° lat. × 0.3125° lon. is higher than that at 4° lat. × 5° lon. by 38%. Similar results are shown for Europe (38% higher) and Australia (37% higher). For sea salt, emissions for North Hemisphere Africa and Southeast Asia increase by 38% and 30%, respectively, from 4° lat. × 5° lon. to 0.25° lat. × 0.3125° lon. The resolution dependence of regional emissions is smaller for BVOCs (within 10% for all regions) than for soil NO_x_ and SSAs.

More figures and tables are available from Peking University Atmospheric Chemistry & Modeling Group, including global and regional monthly totals and spatial distributions from 1980 to 2017 derived based on MERRA-2 at 0.5° lat. × 0.625° lon., as well as respective results from 2014 to 2017 derived based on GEOS-FP at 0.25° lat. × 0.3125° lon., 2° lat. × 2.5° lon., and 4° lat. × 5° lon.

## Technical Validation

### Uncertainty

A major source of uncertainty in our calculated emission data is the use of parameterization as an approximate of the complex processes involved in the emissions of these species^2,6,14^. Parameterization is also sensitive to errors in the inputted meteorological and ancillary data^23,26^.

The parameterization of soil NO_x_ emissions includes a continuous dependence on soil moisture and temperature, a representation of biogeochemistry that induces pulsing of the emissions following dry spells, and a detailed spatial and temporal representation of N-inputs both from chemical/manure fertilizer and atmospheric N-deposition (not included here). Our sensitivity test for 2017 at GEOS-FP 4° lat. × 5° lon. shows that a 1 °C increase in temperature would lead to 5.2% increase in the calculated global total emission, a 10% increase in soil moisture would lead to 15.8% decrease in emission, and a 10% increase in LAI would lead to 1% decrease in emission. Sensitivity tests for other resolutions show similar results.

For sea salt emissions, the strong power law relationship with wind speed and the polynomial relationship with SST mean that errors in wind speed and SST have a significant impact on calculated sea salt emission. Based on our sensitivity test for 2017 at GEOS-FP 4° lat. × 5° lon., a 10% increase in wind speed would lead to 38.4% increase in the calculated global emission total, and a 1 °C increase in SST would lead to 6.7% increase in emission. This is consistent with the evident dependence of calculated emissions to the horizontal resolution of inputted meteorological data. By comparison, an increase in the shape parameter by 10% (from constant 30 to 33) would lead to 0.1% increase in the calculated emissions.

The parameterization of BVOCs emissions involves meteorological (temperature, solar radiation, humidity, wind speed and soil moisture), land cover data (LAI and PFT fractions) and the PFT-specific average emission factor at standard conditions. According to Guenther, *et al*.^14^, uncertainties associated with the global annual emissions of several compounds (isoprene, acetone and acetaldehyde) are about a factor of two while estimates of uncertainties are a factor of three or higher for other compounds here. The average emission factor is the largest contributor to the uncertainty of estimated emission. Uncertainties in land cover and meteorological variables are also important. Wang, *et al*.^42^ showed that an average bias of about 2 °C in temperature is associated with an error in isoprene emissions by ~23% in the Pearl River Delta of China. The error in LAI and its extrapolation to 1980–2004 leads to an additional uncertainty in calculated emissions. Our sensitivity test for 2017 at GEOS-FP 4° lat. × 5° lon. shows that a 1 °C increase in temperature would lead to 12.9% increase in the calculated global total BVOCs, and a 10% increase in LAI would lead to 4.6% increase in emission.

### Comparison with existing emission estimates

Comparisons with existing emission estimates are mainly for our results derived based on MERRA-2, which contain much longer data records than those based on GEOS-FP.

As shown in Table 1, global total above-canopy soil NO_x_ emissions are estimated at 3.3–10 TgN/yr in previous bottom-up studies and at 7.9–16.8 TgN/yr for satellite-based top-down estimates. The Yienger and Levy II^43^ bottom-up algorithm used in many CTM simulations results in global soil NO_x_ emissions of 3.3–7.7 TgN/yr depending on parameters used. Updating upon Yienger and Levy II^43^, Steinkamp and Lawrence^30^ use a new biome type land-cover map and improved emission factors, resulting in an estimate of 8.6 TgN/yr. Hudman, *et al*.^2^ further includes a more physical parameterization that takes into account the pulsing, soil moisture and temperature dependence. Based on Hudman, *et al*.^2^ parameterization and MERRA-2 meteorological data, our calculated soil NO_x_ emissions are 9.5 TgN/yr averaged over 1980–2017, within the (wide) range of values in previous bottom-up and top-down estimates.

Table 2 shows that our global sea salt aerosols emission total (3,560 Tg/yr, based on MERRA-2 for 1980–2017) is in lower end of previous estimates (3,140–10,800 Tg/yr), but is in the middle of the range presented in the IPCC Fifth Assessment Synthesis Report (1,400–6,800 Tg/yr)^44^. It is reduced from the estimate (4,300Tg, based on GEOS-4 winds for 2003) by Jaeglé, *et al*.^6^ by 20%. The decrease is due to the difference in meteorological field data (especially for winds) and some recent updates of sea salt simulation (http://wiki.seas.harvard.edu/geos-chem/index.php/Sea_salt_aerosols#Recent_Updates_to_sea_salt_simulation). In particular, Jaeglé, *et al*.^6^ included one accumulation bin (0.01–0.5 μm) and two coarse mode bins (0.5–4 μm; 4–10 μm), whereas we use one accumulation bin (0.1–0.5 μm) and one coarse bin (0.5–8 μm) here.

The existing estimates of total BVOCs range from 200 to 1,000 TgC/yr depending on the meteorological and vegetation datasets used^15,45^, and our estimate (563 TgC/yr averaged over 1980–2017 based on MERRA-2) is within this range. Table 3 further compares our estimate of global isoprene emission total with others. Our global isoprene emission total (330–345 TgC/yr) is at the lower end of previous bottom-up estimates (303–529 TgC/yr), although it is consistent with those used in various versions of GEOS-Chem (http://wiki.seas.harvard.edu/geos-chem/index.php/Benchmark/GEOS-Chem_12.5.0#GEOS-Chem_Classic_1-month_benchmark). Our isoprene emission total is larger than a recent top-down estimate based on formaldehyde measurements from the Ozone Monitoring Instrument (240 TgC/yr for 2005–2013)^17^, but lower than an earlier top-down estimate (566 TgC/yr for 1996–1997)^46^. The tropical ecosystems are a crucial factor affecting these estimates^17^.

## Data Availability

The official HEMCO code is a collection of FORTRAN-90 routines that are freely available at http://wiki.geos-chem.org/HEMCO. The official HEMCO code with a few necessary changes (based on HEMCO v2.1) to run at the standalone mode are available at Peking University Atmospheric Chemistry & Modeling Group (http://www.phy.pku.edu.cn/~acm/acmProduct.php#NATURAL-EMISSION) and Figshare^29^.

## References

[1] Holland EA, Dentener FJ, Braswell BH, Sulzman JM (1999). Contemporary and pre-industrial global reactive nitrogen budgets. Biogeochemistry.

[2] Hudman RC (2012). Steps towards a mechanistic model of global soil nitric oxide emissions: implementation and space based-constraints. Atmos. Chem. Phys..

[3] Vinken GCM, Boersma KF, Maasakkers JD, Adon M, Martin RV (2014). Worldwide biogenic soil NO_x_ emissions inferred from OMI NO_2_ observations. Atmos. Chem. Phys..

[4] Haywood JM, Ramaswamy V, Soden BJ (1999). Tropospheric Aerosol Climate Forcing in Clear-Sky Satellite Observations over the Oceans. Science.

[5] Ma X, Von Salzen K, Li J (2008). Modelling sea salt aerosol and its direct and indirect effects on climate. Atmos. Chem. Phys..

[6] Jaeglé L, Quinn PK, Bates TS, Alexander B, Lin JT (2011). Global distribution of sea salt aerosols: new constraints from *in situ* and remote sensing observations. Atmos. Chem. Phys..

[7] Sievering H (1992). Removal of sulphur from the marine boundary layer by ozone oxidation in sea-salt aerosols. Nature.

[8] Vogt R, Sander R, von Glasow R, Crutzen PJ (1999). Iodine Chemistry and its Role in Halogen Activation and Ozone Loss in the Marine Boundary Layer: A Model Study. Journal of Atmospheric Chemistry.

[9] Yang, X. *et al*. Tropospheric bromine chemistry and its impacts on ozone: A model study. *Journal of Geophysical Research: Atmospheres* **110**, D23311, 10.1029/2005jd006244 (2005).

[10] Holmes, C. D., Jacob, D. J. & Yang, X. Global lifetime of elemental mercury against oxidation by atomic bromine in the free troposphere. *Geophysical Research Letters* **33**, L20808, 10.1029/2006gl027176 (2006).

[11] Read KA (2008). Extensive halogen-mediated ozone destruction over the tropical Atlantic Ocean. Nature.

[12] Zhu L (2019). Effect of sea salt aerosol on tropospheric bromine chemistry. Atmos. Chem. Phys..

[13] Lamarque JF (2010). Historical (1850–2000) gridded anthropogenic and biomass burning emissions of reactive gases and aerosols: methodology and application. Atmos. Chem. Phys..

[14] Guenther AB (2012). The Model of Emissions of Gases and Aerosols from Nature version 2.1 (MEGAN2.1): an extended and updated framework for modeling biogenic emissions. Geosci. Model Dev..

[15] Sindelarova K (2014). Global data set of biogenic VOC emissions calculated by the MEGAN model over the last 30 years. Atmos. Chem. Phys..

[16] Pfister, G. G. *et al*. Contribution of isoprene to chemical budgets: A model tracer study with the NCAR CTM MOZART-4. *Journal of Geophysical Research: Atmospheres* **113**, D05308, 10.1029/2007jd008948 (2008).

[17] Bauwens M (2016). Nine years of global hydrocarbon emissions based on source inversion of OMI formaldehyde observations. Atmos. Chem. Phys..

[18] Houweling S, Dentener F, Lelieveld J (1998). The impact of nonmethane hydrocarbon compounds on tropospheric photochemistry. Journal of Geophysical Research: Atmospheres.

[19] Taraborrelli D (2012). Hydroxyl radical buffered by isoprene oxidation over tropical forests. Nature Geoscience.

[20] Granier C, Pétron G, Müller J-F, Brasseur G (2000). The impact of natural and anthropogenic hydrocarbons on the tropospheric budget of carbon monoxide. Atmospheric Environment.

[21] Meixner, F. X. & Yang, W. X. In *Dryland Ecohydrology* (eds Paolo D’Odorico & Amilcare Porporato) 233–255 (Springer Netherlands, 2006).

[22] Hudman RC, Russell AR, Valin LC, Cohen RC (2010). Interannual variability in soil nitric oxide emissions over the United States as viewed from space. Atmos. Chem. Phys..

[23] Lin JT (2012). Satellite constraint for emissions of nitrogen oxides from anthropogenic, lightning and soil sources over East China on a high-resolution grid. Atmos. Chem. Phys..

[24] Yan YY, Lin JT, Kuang Y, Yang D, Zhang L (2014). Tropospheric carbon monoxide over the Pacific during HIPPO: two-way coupled simulation of GEOS-Chem and its multiple nested models. Atmos. Chem. Phys..

[25] Yan Y, Lin J, Chen J, Hu L (2016). Improved simulation of tropospheric ozone by a global-multi-regional two-way coupling model system. Atmos. Chem. Phys..

[26] Yu K (2016). Sensitivity to grid resolution in the ability of a chemical transport model to simulate observed oxidant chemistry under high-isoprene conditions. Atmos. Chem. Phys..

[27] Monks PS (2015). Tropospheric ozone and its precursors from the urban to the global scale from air quality to short-lived climate forcer. Atmos. Chem. Phys..

[28] Keller CA (2014). HEMCO v1.0: a versatile, ESMF-compliant component for calculating emissions in atmospheric models. Geosci. Model Dev..

[29] Hongjian W, Jintai L (2020). figshare.

[30] Steinkamp J, Lawrence MG (2011). Improvement and evaluation of simulated global biogenic soil NO emissions in an AC-GCM. Atmos. Chem. Phys..

[31] Cheng W, Tsuruta H, Chen G, Yagi K (2004). N2O and NO production in various Chinese agricultural soils by nitrification. Soil Biology and Biochemistry.

[32] Russell CA, Dunn BW, Batten GD, Williams RL, Angus JF (2006). Soil tests to predict optimum fertilizer nitrogen rate for rice. Field Crops Research.

[33] Linn DM, Doran JW (1984). Effect of Water-Filled Pore Space on Carbon Dioxide and Nitrous Oxide Production in Tilled and Nontilled Soils1. Soil Science Society of America Journal.

[34] Yan, X., Ohara, T. & Akimoto, H. Statistical modeling of global soil NOx emissions. *Global Biogeochemical Cycles* **19**, GB3019 (2005).

[35] Johansson C, Sanhueza E (1988). Emission of NO from savanna soils during rainy season. Journal of Geophysical Research Atmospheres.

[36] Davidson, E. A. Pulses of nitric oxide and nitrous oxide flux following wetting of dry soil: An assessment of probable sources and importance relative to annual fluxes. *Ecological Bulletins* **42**, 149–155 (1992).

[37] Martin RE (1998). Controls on annual emissions of nitric oxide from soils of the Colorado shortgrass steppe. Global Biogeochemical Cycles.

[38] Scholes M, Martin R, Scholes R, Parsons D, Winstead E (1997). NO and N2O emissions from savanna soils following the first simulated rains of the season. Nutrient cycling in Agroecosystems.

[39] Gong, S. L. A parameterization of sea-salt aerosol source function for sub- and super-micron particles. *Global Biogeochemical Cycles* **17**, 10.1029/2003gb002079 (2003).

[40] Yuan H, Dai Y, Xiao Z, Ji D, Shangguan W (2011). Reprocessing the MODIS Leaf Area Index products for land surface and climate modelling. Remote Sensing of Environment.

[41] Ashworth K, Wild O, Hewitt CN (2010). Sensitivity of isoprene emissions estimated using MEGAN to the time resolution of input climate data. Atmos. Chem. Phys..

[42] Wang Y, Jacob DJ, Logan JA (1998). Global simulation of tropospheric O3-NO x -hydrocarbon chemistry: 1. Model formulation. Journal of Geophysical Research: Atmospheres.

[43] Yienger JJ, Levy H (1995). Empirical model of global soil-biogenic NOχ emissions. Journal of Geophysical Research: Atmospheres.

[44] Boucher, O. *et al*. In *Climate change 2013: the physical science basis*. *Contribution of Working Group I to the Fifth Assessment Report of the Intergovernmental Panel on Climate Change* 571–657 (Cambridge University Press, 2013).

[45] Arneth A (2011). Global terrestrial isoprene emission models: sensitivity to variability in climate and vegetation. Atmos. Chem. Phys..

[46] Shim, C. *et al*. Constraining global isoprene emissions with Global Ozone Monitoring Experiment (GOME) formaldehyde column measurements. *Journal of Geophysical Research: Atmospheres* **110**,D24301 (2005).

[47] Miyazaki, K. *et al*. Decadal changes in global surface NO x emissions from multi-constituent satellite data assimilation. *Atmos. Chem. Phys*. **17**, 807–837 (2017).

[48] Stavrakou T (2013). Key chemical NOx sink uncertainties and how they influence top-down emissions of nitrogen oxides. Atmos. Chem. Phys..

[49] Stavrakou, T., Müller, J. F., Boersma, K. F., De Smedt, I. & Van Der A, R. Assessing the distribution and growth rates of NOx emission sources by inverting a 10-year record of NO2 satellite columns. *Geophysical Research Letters* **35**, L10801 (2008).

[50] Jaeglé L, Steinberger L, Martin RV, Chance K (2005). Global partitioning of NO x sources using satellite observations: Relative roles of fossil fuel combustion, biomass burning and soil emissions. Faraday discussions.

[51] Müller J-F, Stavrakou T (2005). Inversion of CO and NOx emissions using the adjoint of the IMAGES model. Atmos. Chem. Phys.

[52] Heald, C. L., Geddes, J. A. & Unger, N. The impact of historical land use change from 1850 to 2000 on secondary particulate matter and ozone. *Atmos. Chem. Phys*. **16**,14997–15010 (2016).

[53] Ganzeveld L (2002). Global soil‐biogenic NOx emissions and the role of canopy processes. Journal of Geophysical Research: Atmospheres.

[54] Müller JF (1992). Geographical distribution and seasonal variation of surface emissions and deposition velocities of atmospheric trace gases. Journal of Geophysical Research: Atmospheres.

[55] Grythe H, Ström J, Krejci R, Quinn P, Stohl A (2014). A review of sea-spray aerosol source functions using a large global set of sea salt aerosol concentration measurements. Atmos. Chem. Phys..

[56] Sofiev, M., Soares, J., Prank, M., de Leeuw, G. & Kukkonen, J. A regional-to-global model of emission and transport of sea salt particles in the atmosphere. *Journal of Geophysical Research: Atmospheres* **116**, D21302 (2011).

[57] Henrot A-J (2017). Implementation of the MEGAN (v2. 1) biogenic emission model in the ECHAM6-HAMMOZ chemistry climate model. Geosci. Model Dev..

[58] Messina P (2016). Global biogenic volatile organic compound emissions in the ORCHIDEE and MEGAN models and sensitivity to key parameters. Atmos. Chem. Phys..

[59] Emmons LK (2010). Description and evaluation of the Model for Ozone and Related chemical Tracers, version 4 (MOZART-4). Geosci. Model Dev..

[60] Young PJ, Arneth A, Schurgers G, Zeng G, Pyle JA (2009). The CO_2_ inhibition of terrestrial isoprene emission significantly affects future ozone projections. Atmos. Chem. Phys..

[61] Müller JF (2008). Global isoprene emissions estimated using MEGAN, ECMWF analyses and a detailed canopy environment model. Atmos. Chem. Phys..

[62] Wiedinmyer C, Tie X, Guenther A, Neilson R, Granier C (2006). Future changes in biogenic isoprene emissions: how might they affect regional and global atmospheric chemistry?. Earth Interactions.

[63] Guenther A (2006). Estimates of global terrestrial isoprene emissions using MEGAN (Model of Emissions of Gases and Aerosols from Nature). Atmos. Chem. Phys..

[64] Naik, V., Delire, C. & Wuebbles, D. J. Sensitivity of global biogenic isoprenoid emissions to climate variability and atmospheric CO2. *Journal of Geophysical Research: Atmospheres* **109**, D06301 (2004).

[65] Levis, S., Wiedinmyer, C., Bonan, G. B. & Guenther, A. Simulating biogenic volatile organic compound emissions in the Community Climate System Model. *Journal of Geophysical Research: Atmospheres* **108**, D21, 4659 (2003).

[66] Guenther A (1995). A global model of natural volatile organic compound emissions. Journal of Geophysical Research: Atmospheres.

